# Supplementary material for: Investigating the influence of physiologically relevant hydrostatic pressure on CHO cell batch culture
Source: Sci Rep. 2021 Jan 8;11:162. doi: 10.1038/s41598-020-80576-8 (PMC7794228; doi:10.1038/s41598-020-80576-8)
Supplement: Supplementary file 1 — Supplementary Information 1. [file 41598_2020_80576_MOESM1_ESM.zip › S1/IgG/Jan17 standard curve/80ugml.pdf]

===

Acq. Operator : SYSTEM Seq. Line : 5
Sample Operator : SYSTEM
Acq. Instrument : HPLC-0039 Location : 5
Injection Date : 1/17/2019 2:09:34 PM Inj : 1
Inj Volume : 100.000 µl
Sequence File : C:\Users\Public\Documents\ChemStation\1\Data\TK_CHO_IgG 2019-01-17 13-50-44\TK_CHO_IgG.S
Method : C:\Users\Public\Documents\ChemStation\1\Data\TK_CHO_IgG 2019-01-17 13-50-44\CHO_IgG_LC.M (Sequence Method)
Last changed : 12/4/2018 1:38:41 PM by SYSTEM

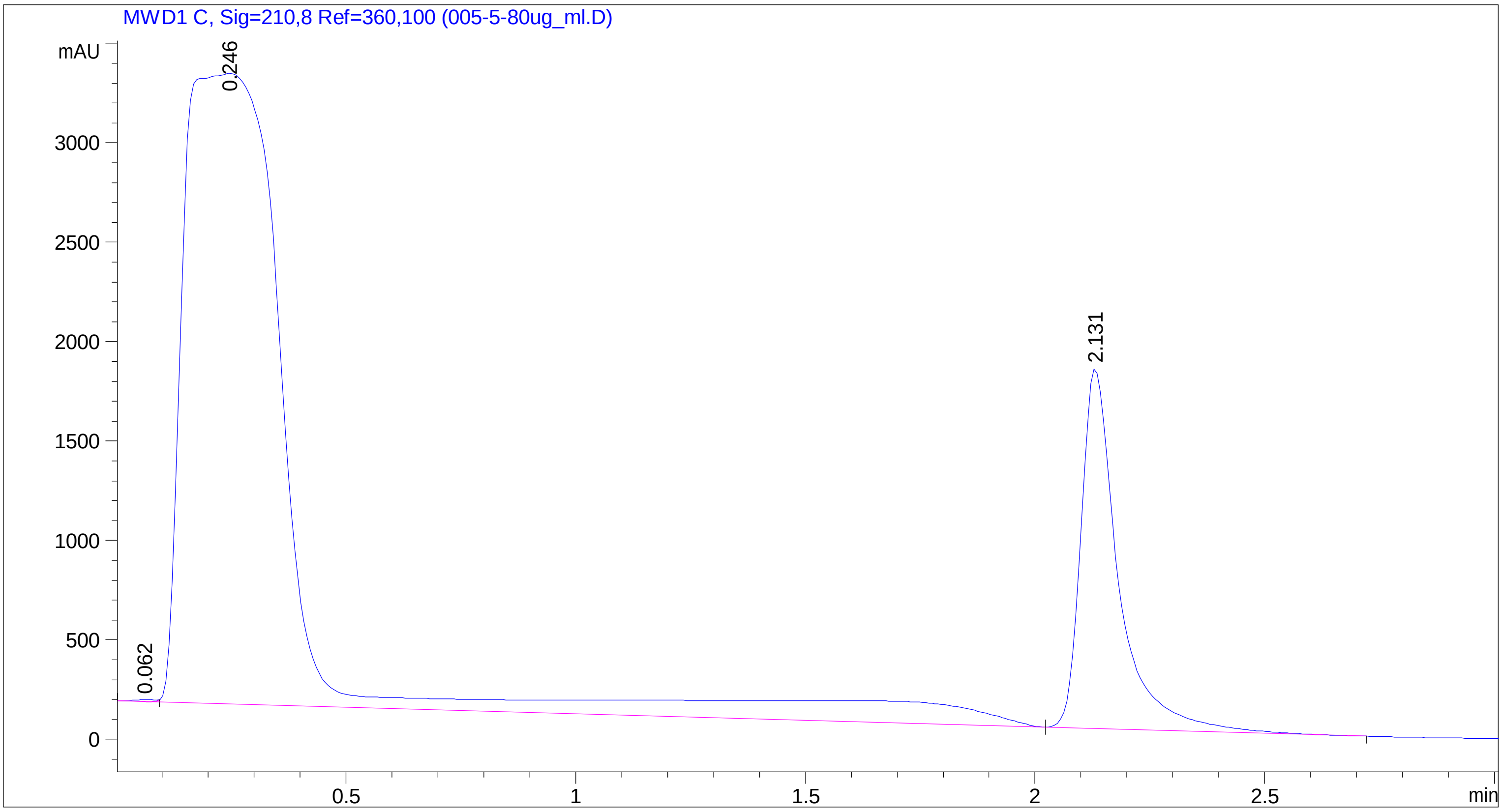

===

Area Percent Report

===

Sorted By : Signal
Multiplier : 1.0000
Dilution : 1.0000
Use Multiplier & Dilution Factor with ISTDs

Signal 1: MWD1 C, Sig=210,8 Ref=360,100

Peak #	RetTime [min]	Type	Width [min]	Area [mAU*s]	Height [mAU]	Area %
1	0.062	BV E	0.0466	31.15117	10.50857	0.0510
2	0.246	VB R	0.2245	5.07015e4	3171.48022	83.0528
3	2.131	BBA	0.0845	1.03147e4	1810.73828	16.8962

Totals : 6.10473e4 4992.72708

===

*** End of Report ***
